# CopraRNA and IntaRNA: predicting small RNA targets, networks and interaction domains

**DOI:** 10.1093/nar/gku359

**Published:** 2014-05-16

**Authors:** Patrick R. Wright, Jens Georg, Martin Mann, Dragos A. Sorescu, Andreas S. Richter, Steffen Lott, Robert Kleinkauf, Wolfgang R. Hess, Rolf Backofen

**Affiliations:** 1 Bioinformatics Group, Department of Computer Science, Albert-Ludwigs-University Freiburg, Georges-Köhler-Allee 106, D-79110 Freiburg, Germany; 2 Genetics and Experimental Bioinformatics, Faculty of Biology, Schänzlestr. 1, D-79104 Freiburg, Germany; 3 Max Planck Institute of Immunobiology and Epigenetics, Stübeweg 51, D-79108 Freiburg, Germany; 4 BIOSS Centre for Biological Signalling Studies, Cluster of Excellence, Albert-Ludwigs-University Freiburg, Germany; 5 Center for non-coding RNA in Technology and Health, University of Copenhagen, Gronnegardsvej 3, DK-1870 Frederiksberg C, Denmark; 6 ZBSA Centre for Biological Systems Analysis, Albert-Ludwigs-University Freiburg, Habsburgerstr. 49, D-79104 Freiburg, Germany

## Abstract

CopraRNA (Comparative prediction algorithm for small RNA targets) is the most recent asset to the Freiburg RNA Tools webserver. It incorporates and extends the functionality of the existing tool IntaRNA (Interacting RNAs) in order to predict targets, interaction domains and consequently the regulatory networks of bacterial small RNA molecules. The CopraRNA prediction results are accompanied by extensive postprocessing methods such as functional enrichment analysis and visualization of interacting regions. Here, we introduce the functionality of the CopraRNA and IntaRNA webservers and give detailed explanations on their postprocessing functionalities. Both tools are freely accessible at http://rna.informatik.uni-freiburg.de.

## INTRODUCTION

In recent years, bacterial small RNAs (sRNAs) have proven to be potent, versatile and important regulators of prokaryotic gene expression (1,2). Furthermore, they are extremely abundant in various prokaryotic genomes (3–7) and due to novel experimental (6,8,9) and computational (10–12) methods on the genomic scale, biologists are struggling with ever increasing magnitudes of sRNA data that can, in many cases, only be harnessed by bioinformatics analyses (i.e. target predictions), preceding wetlab verifications. To make analysis methods accessible to a broad audience, graphical user interfaces (GUIs) are indispensable. Offering such interfaces in a web browser based manner has proven to be useful and intuitive to many users in the past (13–16). The Freiburg RNA Tools webserver aims at supplying an easy to use, free and comprehensive web resource for RNA analysis, also for non-adept users.

Several sRNA target prediction algorithms have been developed in the past (17), and many of them are available as webservers (14,18–21). Here, we highlight that CopraRNA (Comparative prediction algorithm for small RNA targets) (22) and IntaRNA (Interacting RNAs) (23) not only produce more than sound results but also supply postprocessing that greatly aids in the interpretation and evaluation of the results. The tools are accompanied by extensive help pages, and direct help requests are rapidly answered. The results can be viewed in the browser and downloaded for further local analysis or archiving. Furthermore, the source code for both tools is available for download on the Freiburg RNA software page at http://www.bioinf.uni-freiburg.de/Software/.

### CopraRNA AND IntaRNA

**Figure 1.**
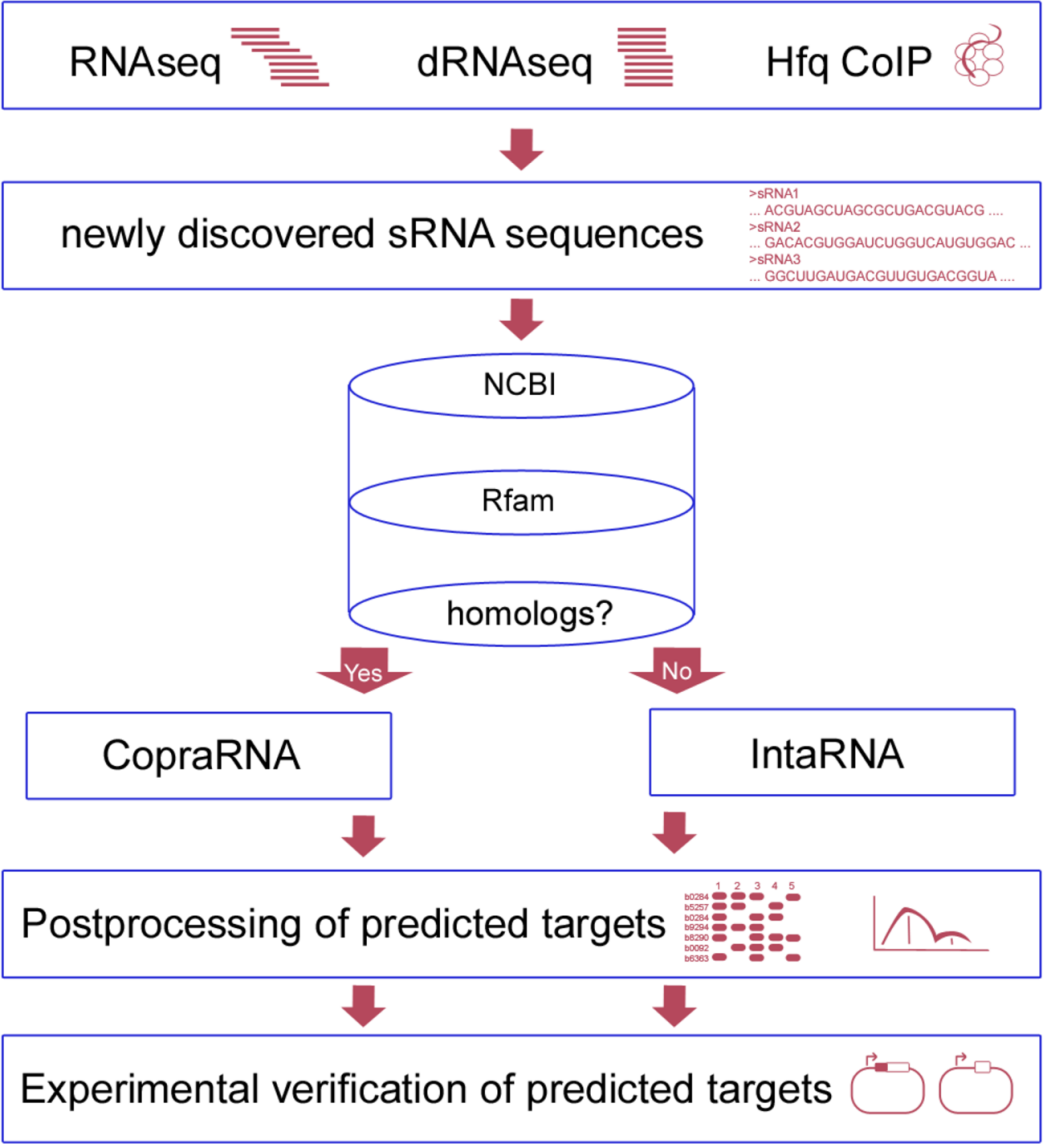
sRNA identification and classification workflow incorporating CopraRNA or IntaRNA. The first box mentions selected experiments that have aided in sRNA identification, i.e. RNAseq (8), dRNAseq (6) or Hfq co-immunoprecipitation (CoIP) (9). The cylinder represents databases that can be queried while looking for sRNA homologs. Examples are NCBI (BLAST) (26) or Rfam (27). The next step is the execution of the actual sRNA target prediction depending on presence of sRNA homologs (CopraRNA) or absence of sRNA homologs (IntaRNA). The final two stages consist of postprocessing and selection of candidates for experimental verification, e.g. by a GFP reporter system (32).

While CopraRNA is a comparative method that constructs a combined sRNA target prediction for a set of given organisms, IntaRNA predicts interactions in single organisms. An exemplary workflow incorporating both tools is given in Figure 1. Employing a statistical model, CopraRNA computes whole genome target predictions by combining whole genome IntaRNA target screens for homologous sRNA sequences from distinct organisms. Individual evolutionary distances between the organisms and the statistical dependencies in the data are accounted for and are corrected within the workflow of the algorithm. IntaRNA predicts interacting regions between two RNA molecules by incorporating the accessibility of both interaction sites and the presence of a seed interaction; both features are commonly observed in sRNA–mRNA interactions (24). IntaRNA, unlike CopraRNA, can also be applied to non-whole genome screens using smaller sets of RNA molecules as input. Thus, it is also applicable to RNA–RNA interaction prediction for eukaryotic systems (25).

## INPUT AND OUTPUT

Input data must be supplied in FASTA format. For CopraRNA, the FASTA file should represent three or more homologous sRNA sequences from distinct organisms. Homologous sRNA sequences may be retrieved from databases such as NCBI via BLAST (26) or from Rfam (27). While only three input sequences are mandatory, we suggest using at least five if available. CopraRNA requires for each sequence, a RefSeq ID of its affiliated organism as FASTA header (see Figure 3A, top left for an example). If several RefSeq IDs correspond to replicons of one organism, any one of these IDs may be supplied. A maximum of eight input organisms is possible. One of these species must be selected as central reference (organism of interest) for postprocessing and annotation.

Currently, ∼2700 organisms are available for CopraRNA and IntaRNA whole genome target predictions and the list is updated on a monthly basis. As previously mentioned, IntaRNA can also compute interactions for smaller sets of RNAs. In this case, the user may supply two FASTA files. For these, all pairwise interactions are computed. Suggested standard parameters for IntaRNA are a seed length (p) of 7, a target folding window size (w) of 150 and a maximum base pair distance (L) of 100 (28).

**Figure 2.**
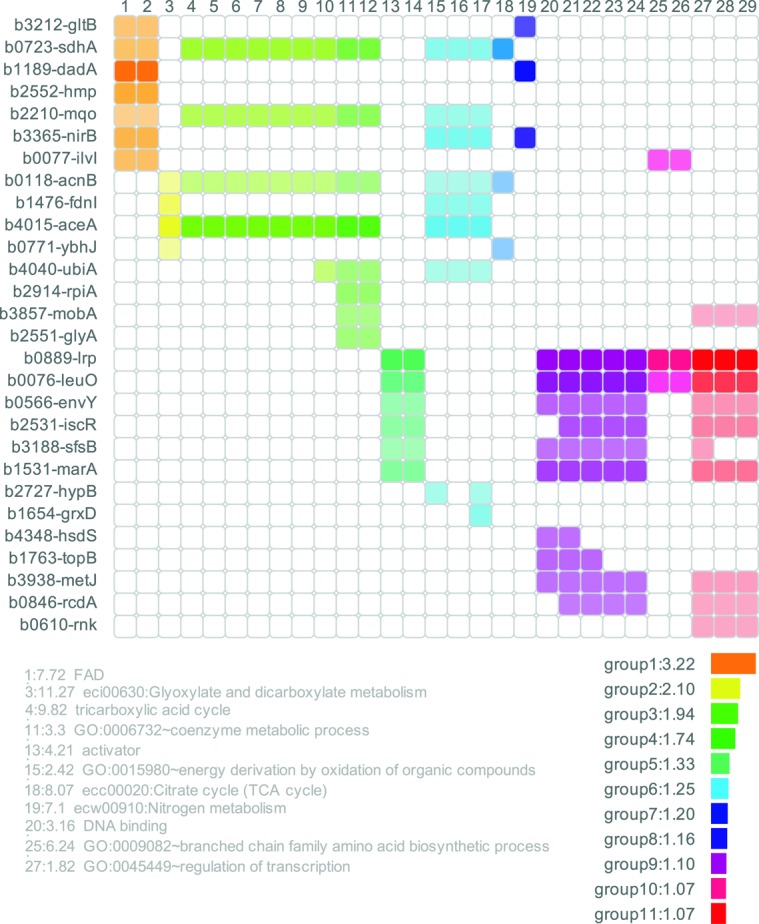
The CopraRNA heatmap shows the targets with a p-value ≤ 0.01 (for IntaRNA the top 50 predicted targets are subjected to the initial functional enrichment), which have homologs in the organism of interest and are functionally enriched. All members of clusters with a DAVID enrichment score ≥ 1.0 are shown in a specific color. Each row represents a gene and each column a specific functional term. If the gene can be assigned to a term, the corresponding square is colored. If no assignment was made, the square remains white. Closely related terms are assigned to a cluster and have the same color. The opacity of the color depends on the p-value of the CopraRNA prediction. A more intense color represents a more significant p-value. The ‘fold enrichment’ is given in front of the term descriptions. It represents the enrichment of a term in the prediction group in relation to the whole prediction background (e.g. a term with an enrichment of 10 contains 10 times more genes belonging to the respective term than the background). The enrichment scores give a measure of the biological significance of the cluster. The DAVID enrichment score for a cluster is the log transformed geometric mean of all enrichment p-values from the terms belonging to the respective cluster. A higher score represents a more statistically significant enrichment. The individual p-values for the terms are calculated by a modified Fisher's exact test. The length of the bars next to the groups of enriched genes corresponds to the size of the enrichment score. The publication on the DAVID webserver suggests to investigate clusters with an enrichment score of ≥ 1.3 while also pointing out that clusters with lower enrichment scores must not necessarily be discarded and may also contain useful information (33). This specific heatmap represents the enrichment output for the enterobacterial (here *Escherichia coli*) sRNA FnrS. Due to space reasons only one term for each cluster is shown.

**Figure 3.**
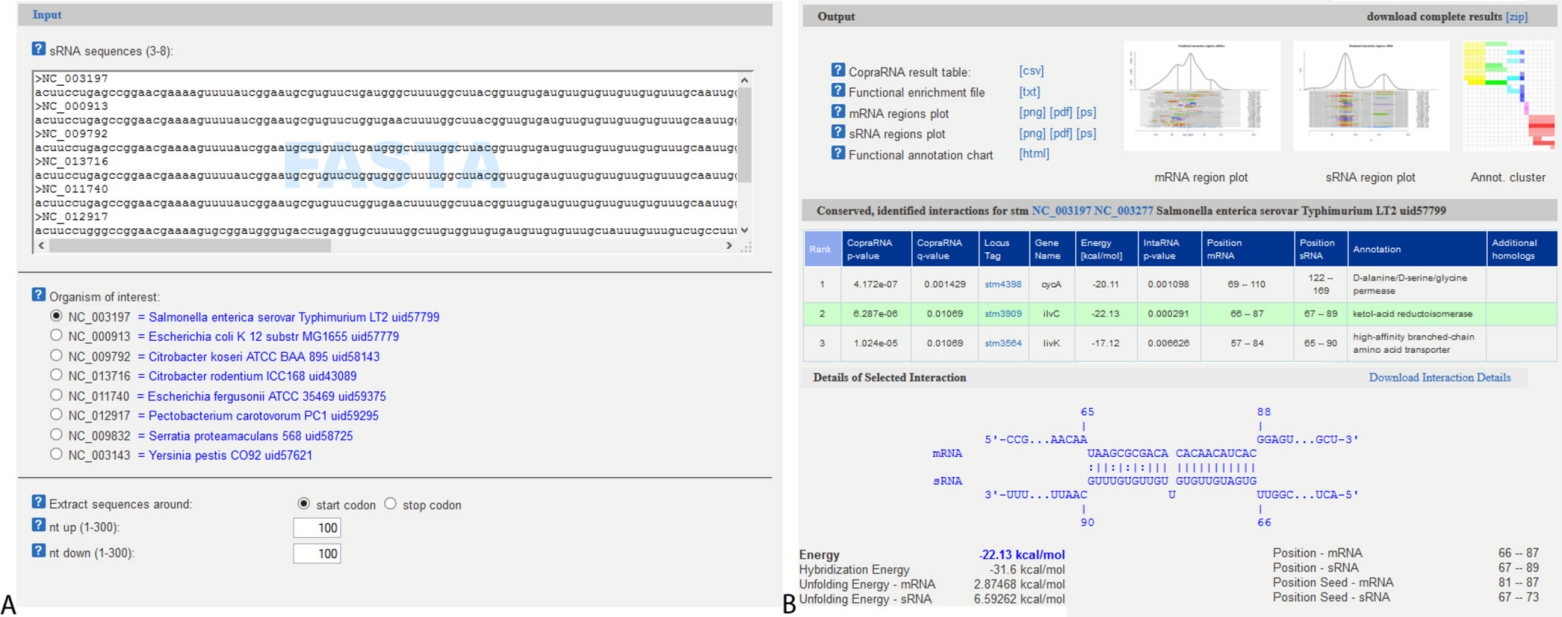
CopraRNA webserver input (**A**) and output (**B**) page for the sRNA GcvB. The FASTA file may be pasted or uploaded to the webserver. Upon insertion of the sequences, the webserver automatically displays the RefSeq IDs’ organism affiliations (blue text in (A)). The output page contains a visualization of the primary result table, the interaction as predicted by IntaRNA and the interacting region plots within the sRNA and mRNA. Furthermore, the functional enrichment is visualized as interactive heatmap.

Both webservers provide the top 100 predictions of the respective methods as primary result table. Furthermore, the core results of the algorithms are accompanied by extensive postprocessing that aids interpretation and condensation of the result tables. For whole genome target predictions, CopraRNA and IntaRNA include automatic functional enrichment (29) of the top predicted targets and visualization of putative interacting regions within the sRNA and the mRNA. As a new feature of the webserver, the functionally enriched terms are represented within a heatmap, allowing ‘at a glance’ conclusions for target networks (see Figure 2 for an example). These results can guide the user while constructing functional networks and characterizing target binding mechanisms of a given sRNA. For users interested in the entire results, the corresponding job is available for download as compressed archive. Sample input and output pages for CopraRNA are displayed in Figure 3. Both tools’ source code is also available for download and local installation from the Freiburg RNA webserver download page.

## METHODS

CopraRNA utilizes IntaRNA to calculate single organism whole genome target predictions. IntaRNA predictions are computed for each sRNA-organism pair participating in the analysis. These individual predictions are the basis for the comparative model. In order to combine target predictions for homologous genes from distinct organisms, IntaRNA p-values are computed from the IntaRNA energy scores for each putative target with an energy score ≤ 0. Transforming energy scores to p-values is achieved by fitting generalized extreme value distributions to the IntaRNA energy scores. Using the resulting equations for each individual whole genome target prediction, p-values can be calculated for each putative target. In the following, the DomClust (30) algorithm is applied in order to cluster homologous genes. The clustering is based on the amino acid sequences of the organisms’ protein coding genes. These clusters are then used to calculate a combined CopraRNA p-value for each cluster of homologous genes by employing Hartung's method for the combination of dependent p-values (31). Conveniently, it not only allows to account for the overall dependency within the data, but also incorporates the possibility to weight individual p-values. This is important, as the organisms participating in the analysis can usually not be regarded as equidistant. Closer organisms are consequently down weighted. Excessive influence of outliers is corrected for by applying a root function to the weights. The final set of CopraRNA p-values is employed for q-value calculation. The q-values give an estimate of the false discovery rate of the target prediction. More detailed algorithmic explanations on CopraRNA and IntaRNA are given in the original publications (22,23).

## POSTPROCESSING AND PREDICTION QUALITY ESTIMATION

The benchmarking of CopraRNA showed that some predictions are more reliable (e.g. GcvB, RyhB, FnrS) than others (e.g. ArcZ) (22). On behalf of a reduced experimental (32) workload it is preferable to have a measure for the reliability of each individual prediction. Here the q-value and the postprocessing outputs provide guidance. A strong functional enrichment signature, pointing to a specific group of genes or a specific pathway, has proven to be a reliable signal for a meaningful prediction. However, functional enrichments are not always present. This may be due to low prediction quality, but it can also be caused by a lack of annotation for the organism of interest or its absence in the DAVID knowledge base (33).

In these cases the user may opt to choose the organism with the best available annotation as organism of interest. If this proves ineffective, the user should resort to the q-value distribution and the interaction domain plots. A slowly growing q-value, i.e. a relatively high number of predictions with a q-value ≤ 0.5, is a hallmark of a more reliable prediction, especially if the interaction plots show distinct clustered interaction regions for the sRNA and mRNA homologs. A random distribution of the interaction sites in the mRNAs and/or sRNA homologs argues against a reliable prediction.

## JOB ARCHIVING

Upon submission, a unique ID, which is only known by the submitting user, is automatically assigned to each job. This ID can be used to recall the results of a specific job at any time within the storage period. The Freiburg RNA webserver stores all computed results for 30 days. Within this time, selected results or the entire job directory may be downloaded for local archiving by the user. Online archiving within the 30 day period is aided by the possibility of setting job specific descriptions.

## PRIOR APPLICATION AND EVALUATION

The predictive performance of CopraRNA and IntaRNA was previously evaluated on an extensive benchmarking dataset of 101 experimentally verified sRNA and target pairs from 18 enterobacterial sRNAs (22). They were compared to each other and to RNApredator (19) and TargetRNA (18). Both tools from the Freiburg RNA webserver outperformed the other tools in predictive accuracy. Furthermore, CopraRNA was compared to experimental target prediction by micro arrays. Strikingly, it showed similar predictive quality with respect to the abundance of correctly predicted targets (22). From the CopraRNA benchmark predictions, 23 previously unreported, putative sRNA targets were selected for experimental verification. From these, 17 were verified (22). This represents a success rate of ∼74%. CopraRNA has also been successfully applied in studies on non-enterobacterial species. These include investigations of the sRNAs PsrR1 from *Synechocystis* *sp.* PCC6803 and AbcR1 from *Agrobacterium tumefaciens* (unpublished data). Beside many other studies, computational predictions with IntaRNA enabled the identification of two novel targets of the cyanobacterial sRNA Yfr1 (34) and aided in finding that the archaeal sRNA162 targets both *cis*- and *trans*-encoded mRNAs via two distinct domains (35).

## IMPLEMENTATION

The Freiburg RNA webserver is based on Apache Tomcat Java Server Pages (JSP) to enable a high server-side performance for input validation, job execution and retrieval, and dedicated pre- and postprocessing. Javascripting is used to provide an intuitive and interactive user interface on the client side. The tools provided by the Freiburg RNA webserver are run on a dedicated computing cluster with up to 480 CPUs, depending on the workload. Jobs are automatically queued and started via Sun Grid Engine to ensure a balanced and fast job processing given the varying execution requirements of the different tools provided. An automatic emailing system informs the user upon job completion if an email address (optional) is provided upon submission.

## References

[1] Storz G., Vogel J., Wassarman K.M. (2011). Regulation by small RNAs in bacteria: expanding frontiers. Mol. Cell.

[2] Gottesman S., Storz G. (2011). Bacterial small RNA regulators: versatile roles and rapidly evolving variations. Cold Spring Harbor Perspect. Biol..

[3] Dugar G., Herbig A., Forstner K.U., Heidrich N., Reinhardt R., Nieselt K., Sharma C.M. (2013). High-resolution transcriptome maps reveal strain-specific regulatory features of multiple Campylobacter jejuni isolates. PLoS Genet..

[4] Mitschke J., Georg J., Scholz I., Sharma C.M., Dienst D., Bantscheff J., Voß B., Steglich C., Wilde A., Vogel J. (2011). An experimentally anchored map of transcriptional start sites in the model cyanobacterium *Synechocystis* sp. PCC6803. Proc. Natl. Acad. Sci. U.S.A..

[5] Georg J., Hess W.R. (2011). Regulatory RNAs in cyanobacteria: developmental decisions, stress responses and a plethora of chromosomally encoded cis-antisense RNAs. Biol. Chem..

[6] Sharma C.M., Hoffmann S., Darfeuille F., Reignier J., Findeiß S., Sittka A., Chabas S., Reiche K., Hackermüller J., Reinhardt R. (2010). The primary transcriptome of the major human pathogen *Helicobacter pylori*. Nature.

[7] Kroger C., Colgan A., Srikumar S., Handler K., Sivasankaran S.K., Hammarlof D.L., Canals R., Grissom J.E., Conway T., Hokamp K. (2013). An infection-relevant transcriptomic compendium for Salmonella enterica Serovar Typhimurium. Cell Host Microbe.

[8] Wang Z., Gerstein M., Snyder M. (2009). RNA-Seq: a revolutionary tool for transcriptomics. Nat. Rev. Genet..

[9] Zhang A., Wassarman K.M., Rosenow C., Tjaden B.C., Storz G., Gottesman S. (2003). Global analysis of small RNA and mRNA targets of Hfq. Mol. Microbiol..

[10] Voß B., Georg J., Schön V., Ude S., Hess W.R. (2009). Biocomputational prediction of non-coding RNAs in model cyanobacteria. BMC Genomics.

[11] Babski J., Tjaden B., Voss B., Jellen-Ritter A., Marchfelder A., Hess W.R., Soppa J. (2011). Bioinformatic prediction and experimental verification of sRNAs in the haloarchaeon Haloferax volcanii. RNA Biol..

[12] Amman F., Wolfinger M.T., Lorenz R., Hofacker I.L., Stadler P.F., Findeiss S. (2014). TSSAR: TSS annotation regime for dRNA-seq data. BMC Bioinformatics.

[13] Lorenz R., Bernhart S.H., Höner Zu Siederdissen C., Tafer H., Flamm C., Stadler P.F., Hofacker I.L. (2011). ViennaRNA Package 2.0. Algorithms Mol. Biol..

[14] Smith C., Heyne S., Richter A.S., Will S., Backofen R. (2010). Freiburg RNA Tools: a web server integrating IntaRNA, ExpaRNA and LocARNA. Nucleic Acids Res..

[15] Sorescu D.A., Möhl M., Mann M., Backofen R., Will S. (2012). CARNA—alignment of RNA structure ensembles. Nucleic Acids Res.

[16] Lange S.J., Alkhnbashi O.S., Rose D., Will S., Backofen R. (2013). CRISPRmap: an automated classification of repeat conservation in prokaryotic adaptive immune systems. Nucleic Acids Res..

[17] Backofen R., Hess W.R. (2010). Computational prediction of sRNAs and their targets in bacteria. RNA Biol..

[18] Tjaden B., Goodwin S.S., Opdyke J.A., Guillier M., Fu D.X., Gottesman S., Storz G. (2006). Target prediction for small, noncoding RNAs in bacteria. Nucleic Acids Res..

[19] Eggenhofer F., Tafer H., Stadler P.F., Hofacker I.L. (2011). RNApredator: fast accessibility-based prediction of sRNA targets. Nucleic Acids Res..

[20] Ying X., Cao Y., Wu J., Liu Q., Cha L., Li W. (2011). sTarPicker: a method for efficient prediction of bacterial sRNA targets based on a two-step model for hybridization. PLoS One.

[21] Cao Y., Zhao Y., Cha L., Ying X., Wang L., Shao N., Li W. (2009). sRNATarget: a web server for prediction of bacterial sRNA targets. Bioinformation.

[22] Wright P.R., Richter A.S., Papenfort K., Mann M., Vogel J., Hess W.R., Backofen R., Georg J. (2013). Comparative genomics boosts target prediction for bacterial small RNAs. Proc. Natl. Acad. Sci. U.S.A..

[23] Busch A., Richter A.S., Backofen R. (2008). IntaRNA: efficient prediction of bacterial sRNA targets incorporating target site accessibility and seed regions. Bioinformatics.

[24] Richter A.S., Backofen R. (2012). Accessibility and conservation: general features of bacterial small RNA-mRNA interactions?. RNA Biol..

[25] Starczynowski D.T., Morin R., McPherson A., Lam J., Chari R., Wegrzyn J., Kuchenbauer F., Hirst M., Tohyama K., Humphries R.K. (2011). Genome-wide identification of human microRNAs located in leukemia-associated genomic alterations. Blood.

[26] Altschul S.F., Gish W., Miller W., Myers E.W., Lipman D.J. (1990). Basic local alignment search tool. J. Mol. Biol..

[27] Burge S.W., Daub J., Eberhardt R., Tate J., Barquist L., Nawrocki E.P., Eddy S.R., Gardner P.P., Bateman A. (2013). Rfam 11.0: 10 years of RNA families. Nucleic Acids Res..

[28] Lange S.J., Maticzka D., Möhl M., Gagnon J.N., Brown C.M., Backofen R. (2012). Global or local? Predicting secondary structure and accessibility in mRNAs. Nucleic Acids Res..

[29] Jiao X., Sherman B.T., Huang D.W., Stephens R., Baseler M.W., Lane H.C., Lempicki R.A. (2012). DAVID-WS: a stateful web service to facilitate gene/protein list analysis. Bioinformatics.

[30] Uchiyama I. (2006). Hierarchical clustering algorithm for comprehensive orthologous-domain classification in multiple genomes. Nucleic Acids Res..

[31] Hartung J. (1999). A note on combining dependent tests of significance. Biom. J..

[32] Corcoran C.P., Podkaminski D., Papenfort K., Urban J.H., Hinton J.C.D., Vogel J. (2012). Superfolder GFP reporters validate diverse new mRNA targets of the classic porin regulator, MicF RNA. Mol. Microbiol..

[33] Huang D.W., Sherman B.T., Lempicki R.A. (2009). Systematic and integrative analysis of large gene lists using DAVID bioinformatics resources. Nat. Protoc..

[34] Richter A.S., Schleberger C., Backofen R., Steglich C. (2010). Seed-based IntaRNA prediction combined with GFP-reporter system identifies mRNA targets of the small RNA Yfr1. Bioinformatics.

[35] Jäger D., Pernitzsch S.R., Richter A.S., Backofen R., Sharma C.M., Schmitz R.A. (2012). An archaeal sRNA targeting *cis*- and *trans*-encoded mRNAs via two distinct domains. Nucleic Acids Res..

